# Oral narrative discourse in schoolchildren with Autism Spectrum Disorder

**DOI:** 10.1590/2317-1782/e20250042en

**Published:** 2026-04-10

**Authors:** Luísa Stefano Santos, Patrícia Aparecida Zuanetti, Julia Branco Zulian, Matheus Francoy Alpes, Angela Cristina Pontes-Fernandes, Marisa Tomoe Hebihara Fukuda

**Affiliations:** 1 Departamento de Psicologia, Faculdade de Filosofia, Ciências e Letras de Ribeirão Preto – FFCLRP, Universidade de São Paulo – USP - Ribeirão Preto (SP), Brasil.; 2 Hospital das Clínicas de Ribeirão Preto, Faculdade de Medicina de Ribeirão Preto, Universidade de São Paulo – USP - Ribeirão Preto (SP), Brasil.; 3 Departamento de Formação Específica de Fonoaudiologia, Instituto de Saúde de Nova Friburgo, Universidade Federal Fluminense – INSF/UFF - Nova Friburgo (RJ), Brasil.; 4 Departamento Ciências da Saúde, Faculdade de Medicina de Ribeirão Preto, Universidade de São Paulo – USP - Ribeirão Preto (SP), Brasil.

**Keywords:** Autism Spectrum Disorder, Narration, Child Language, Child Development, Protective Factors

## Abstract

**Purpose:**

To analyze the characteristics of oral narrative discourse in children diagnosed with autism spectrum disorder (ASD).

**Methods:**

The study included schoolchildren aged 6 to 10 years, divided into two groups: ASD group (n = 26), with oral children diagnosed with ASD; and control group (n = 26), with typically developing children. Sociodemographic data were collected through questionnaires applied to parents/guardians, and the schoolchildren were evaluated regarding intellectual estimation and oral narrative discourse (partial retelling, total retelling, and story comprehension); the story was read aloud to them.

**Results:**

The schoolchildren belonged to socioeconomic classes A/B, and most mothers had a bachelor’s degree. No schoolchild was classified as intellectually disabled. The oral narrative instrument identified a difference between the groups in the partial retelling test; the ASD group performed worse. There was no difference in total retelling or oral comprehension. Regarding the morphosyntax used in constructing narrative sentences, a difference was identified in the use of nouns and prepositions, with poorer performance in the ASD group. No significant differences were observed in the use of other morphosyntactic categories.

**Conclusion:**

Schoolchildren with ASD, in favorable sociodemographic conditions, present oral narrative performance similar to that of typically developing children, with occasional differences.

## INTRODUCTION

Oral narrative is a form of intentional communication and planned discursive thought to share experiences ordered in time, with causality and coherence^(1)^. This skill plays a crucial role in organizing experience, facilitating social interaction and knowledge construction. It is considered a complex cognitive and linguistic skill^(2-3)^, requiring the integrity of executive functions^(4-5)^ and mastery of semantic and pragmatic skills^(6)^.

Although the definition of narrative is controversial, there is general consensus that it refers to a sequence of events in time^(7-8)^. Thus, narrative discourse can be defined as a complex cognitive task, involving the integration of information, cognitive skills, and the use of accumulated world knowledge^(9)^. In other words, narrative would be the ability to think, communicate, and share experiences^(10)^.

Narrative production involves the integration of two main components: one linguistic and the other cognitive. The linguistic component, linked to the microstructure of the narrative, encompasses aspects such as phonology, morphosyntax, and semantics, being responsible for the content of the story. The cognitive component, related to the macrostructure, involves higher-order cognitive functions such as executive functions – e.g., the ability to inhibit, plan, and organize, to maintain the theme and the logical and temporal sequence of events, and to understand causal relationships between them^(11-12)^. These elements interact to ensure the cohesion and coherence of the narrative discourse^(13)^.

The development of textual coherence is a gradual process that reflects the maturation of the child's linguistic, cognitive, and discursive skills. As they advance in their linguistic repertoire and their ability to organize thoughts and ideas, they begin to structure narratives more cohesively, with greater connection between events and clarity in thematic progression. Researchers in the field have described this process as a trajectory that can be observed through levels of performance, ranging from disorganized narratives, with difficulties in maintaining the theme and characters, to well-structured productions, with logically linked events and outcomes compatible with the developed plot. This perspective allows us to understand and evaluate narrative coherence as a developing skill progressively refined throughout childhood^(14)^. Instruments aimed at evaluating the macrostructural aspects of the narrative analyze elements such as setting, theme, plot development, conflicts, and their resolution^(13-14)^.

Alongside the development of coherence, textual cohesion plays an essential role in narrative organization, referring to the linguistic mechanisms that connect the parts of the text to each other. Cohesion corresponds to the microstructure of the narrative, formed by resources such as connectives, pronouns, repetitions, and lexical substitutions that contribute to the continuity and linking of ideas. The appropriate use of these elements presents a fluid text, facilitating overall comprehension and reinforcing its coherence^(15)^. Although cohesion alone does not guarantee textual coherence, the two are closely related, since cohesive resources function as fundamental linguistic tools for the construction of interpretable and well-structured narratives^(14)^. Microstructural aspects are analyzed through measures such as the total number of words, varied vocabulary, use of conjunctions, communicative units, and lexical diversity, among other linguistic resources^(13)^.

Oral narrative is one of the most elaborate levels of language in either comprehension or production. Therefore, individuals with impairments at earlier levels — e.g., in the development of linguistic aspects, such as semantic, pragmatic, and others — are expected to perform poorly in it^(6,10)^.

The basic impairments of individuals with autism spectrum disorders (ASD) are persistent deficits in two main areas: difficulties in social communication skills (pragmatic aspect of language and social cognition) in multiple contexts; and restricted and repetitive patterns of behavior, interests, or activities, hyposensitivity or hypersensitivity to sensory stimuli (e.g., sounds and textures), and stereotypies^(16)^. In addition to these basic characteristics, children with ASD may also have more global linguistic and cognitive alterations as possible specifiers^(16)^.

Oral narrative, usually elicited by visual stimuli, has been studied in individuals with ASD^(3,5,17-23)^. However, the findings are varied and sometimes inconsistent. The diversity in findings is due to the diversity of samples evaluated (varying levels of ASD severity, association with other comorbidities such as intellectual disability, sample age, changes in diagnostic criteria for ASD in recent years, and so forth).

In general, studies have identified impairments in oral narrative comprehension and production skills^(17-18)^; difficulty in identifying, understanding, and describing the emotions of the characters; less frequent dialogic interactions between characters in the story; impaired comprehension of metaphors and metonymies^(19-20)^; reduced speech rate and more hesitations^(3)^; shorter and grammatically less complex narratives^(5)^; omission of key story components^(21)^; better performance in narrative production when using sequences of pictures, instead of a single picture^(22)^; among other findings. But, at the same time, these studies^(3,5,22)^ found similar performance in other variables (e.g., the number of words related to temporal relationships, the number of mental states produced, story comprehension and production, among others) between typical children and children with ASD.

Given the relevance of narratives to social and communicative development and the specific challenges faced by individuals with ASD, studying such a skill in this group is fundamental. Such research not only provides a more detailed understanding of the impacts of ASD on communication but also supports the development of intervention strategies for more effective narrative skills, helping to strengthen social interactions and improve the quality of life of individuals with ASD and their families. This study aimed to analyze the characteristics of the narrative discourse of children diagnosed with ASD.

## METHODS

### Study model and ethical considerations

This is a cross-sectional observational study, conducted in accordance with the guidelines of the Brazilian National Research Ethics Committee (CONEP), submitted to and approved by the Research Ethics Committee of FFCLRP - USP (CAAE no. 40972920.5.0000.5407). All parents/guardians of the children evaluated were informed about the nature of the research and instructed to sign an informed consent form.

### Sample selection and collection site

The sample size was established by convenience, based on estimates of the number of subjects available at the collection sites (i.e., children who met the study inclusion criteria). The children in the ASD group were recruited from a multidisciplinary clinic specializing in the care of children with ASD, where they attended some type of therapeutic intervention. Approximately 50 patients from the clinic, diagnosed with ASD, aged 6 to 10 years, who met the inclusion criteria, were invited to participate in the study, of which only 26 parents/guardians authorized their children's participation.

The children in the control group (CG) were selected from a private school so that their ages and sexes matched those of the ASD group. The school provided the list of enrolled students for the selection of children who met the research criteria for the CG and who matched the ages and sexes of the ASD group. If more than one child was eligible, a draw was held to determine which one would be invited to participate in the research first. If their parent/guardian was not interested, the second child matched was invited.

The inclusion criteria were children aged 6 to 10 years. For the ASD group, the criteria were children with a medical and multidisciplinary diagnosis of ASD, according to the DSM-5-TR criteria^(16)^; they also had to have oral skills – i.e., producing simple and complex intelligible sentences, with adequate morphosyntactic structure, and without moderate or severe phonological alterations. The CG included only children who were not enrolled in special education programs or with curricular adaptations/individualized teaching programs, which excluded those with a diagnosis of ASD, intellectual disability, syndromes, or other neurodevelopmental conditions.

The exclusion criteria were refusal, non-performance, or interruption in the application of the instruments. Specifically, the CG excluded children with any medical or multidisciplinary diagnosis of neurodevelopmental disorder and those using medication with cognitive effects. However, a history of multidisciplinary therapies and being on non-pharmacological therapy at the time of assessment were not inclusion/exclusion criteria for either group.

The final study sample consisted of 52 children aged 6 to 10 years, divided into:

- ASD group (n = 26; mean age 7.2; SD: 1.3; 20 males), comprising oral children with ASD.
- CG (n = 26; mean age 7.1; SD: 1.3; 20 males), comprising typically developing children, matched by age and sex to the ASD group.

### Data collection instruments and procedures

Both at the clinic and at school, the children were assessed individually in rooms with adequate lighting and without competing external noise that could compromise their performance. Only the child and the examiner were present in the room. Except for the intellectual estimation assessment, which was carried out by a psychologist, all other instruments were applied by a speech-language-hearing pathologist and recorded using Audacity^®^ software, using a microphone so that the child's response could be analyzed later.

The following instruments were used with the parents/guardians:

- Initial interview: A brief structured questionnaire answered by the parents/guardians to collect information regarding the child's history. It aimed to apply/confirm the inclusion/exclusion criteria and to characterize the sample.
- Brazilian Economic Classification Criteria (ABEP)^(23)^: This instrument classifies the socioeconomic level of the research subject's family. It is answered by the parent/guardian, verifying the number of specific items in the family's residence, the householder’s education level, and their access to public services. The family’s socioeconomic level was scored and classified according to the answers and used to characterize the sample.

The following instruments were used with the children:

- Raven's Colored Progressive Matrices^(24)^, a nonverbal measure of intelligence. The colored scale is specifically designed for children, individuals with communication difficulties, and those with atypical development, by minimizing the influence of linguistic and cultural factors on performance. The test consists of a series of visual problems presented in matrix format, in which the individual must identify the pattern or logic governing the sequence and select the correct alternative to complete it. It was applied individually, following the technical guidelines of the test manual. This study used its results to characterize the sample, and the data were presented as a categorical variable (performance classification according to test norms).
- Oral Narrative Discourse (DNOI)^(4)^, an assessment instrument that evaluates language at the discursive level, being useful for evaluating subjects with language difficulties and executive dysfunction. It consists of three subtasks: partial retelling of a narrative (the child was informed that they would hear a short story, and that after each paragraph, they should retell what they had just heard); complete retelling of the same narrative (the entire text was read to the child, who, at the end, had to retell the story in their own words); and literal and inferential discursive comprehension (the child was asked to create a title for the narrative heard and answer to questions about the story). This task does not have visual support. In all three subtasks, the evaluator reads the paragraphs or texts to the children and asks the questions orally. The answers must also be given orally.

It was applied individually, following the technical guidelines of the test manual. The responses were recorded and subsequently analyzed. The quantitative performance results were analyzed based on the calculation of the z-score (transformation of the raw score according to the child's age and type of school, as suggested by the instrument). Based on the z-score, the child's performance in the various tasks was also classified as adequate (z-score greater than -0.99) or impaired (z-score equal to or less than -1.0).

- Morphosyntactic content of the discourse: Morphosyntactic aspects in the narrative sample were analyzed according to parameters described by means of the “Average Sentence Value” analysis^(25)^. The latter considered the first five sentences spoken by the child in the DNOI complete retelling test, which were evaluated and scored according to the syntactic and lexical elements used, taking into account their complexity: nouns and verbs (considered the first to appear in language acquisition and giving meaning to the sentence) had a weight of 2 (i.e., the number of nouns and verbs used by the child was multiplied by 2); adverbs, adjectives, prepositions, conjunctions, pronouns, and articles had a weight of 4 (i.e., the number of words in these categories emitted by the child was multiplied by 4). The results of the child’s numerical performance were analyzed based on the weight corresponding to each syntactic element (2 or 4, as described above).

### Statistical data analysis

The study used descriptive statistics to characterize the groups and, for inference, employed the two-proportion z-test (comparison of some medical history data and qualitative variables/performance classifications in the various tests used) and the Mann-Whitney test (comparison of quantitative variables, i.e., the z-score). The significance level in both tests was set at α = 0.05.

## RESULTS

Regarding education level, 20 children (76.9%) in the ASD group were enrolled in elementary school (13 in the 1^st^ grade, three in the 2^nd^ grade, three in the 3^rd^ grade, and two in the 5^th^ grade). In the CG, 25 children (96.1%) attended elementary school (14 in the 1^st^ grade, five in the 2^nd^ grade, four in the 3^rd^ grade, and two in the 4^th^ grade). The parents/guardians reported two cases of grade retention in both groups.

Table 1 presents sociodemographic variables, including maternal education and socioeconomic classification. There was a predominance of mothers with higher education in both groups, as well as a greater concentration of families in higher classes (A and B).

**Table 1:** Characterization of the sample regarding maternal education and socioeconomic classification (Brazilian Economic Classification Criteria – ABEP)^(17)^

		**ASD group**	**Control Group**
		**Abs. Freq. (%)**	**Abs. Freq. (%)**
**Maternal Education**	Middle school	1 (3.8%)	0 (0%)
	High school	2 (7.6%)	2 (7.6%)
	Higher education, incomplete	4 (15.3%)	1 (3.8%)
	Bachelor’s degree	16 (61.5%)	15 (57.6%)
	Postgraduate	3 (11.5%)	8 (30.7%)
**Economic Classification**	Class A	9 (34.6%)	13 (50%)
	Class B1	6 (23%)	5 (19.2%)
	Class B2	10 (38.4%)	6 (23%)
	Class C1	1 (3.8%)	2 (7.6%)

**Caption:** ASD = autism spectrum disorder; Abs. Freq. (%) = Data presented in the table by absolute frequency and percentage in parentheses. Source: The authors

The average age at diagnosis in the ASD group was 4.0 years (SD: 1.7), with a minimum age of 2 years and a maximum age of 8 years. These data reveal a wide variation in the age of diagnosis, indicating that some children were identified and referred for intervention in early childhood, while others, possibly with milder manifestations of ASD, received a later diagnosis.

The ASD group had a higher prevalence of a history of therapeutic intervention. All children in this group (n = 26; 100%) had a history of therapy (speech-language-hearing therapy, psychology, and/or occupational therapy). In the CG, only six children (23%) reported some type of intervention. At the time of data collection, all children in the ASD group were undergoing active therapeutic follow-up in at least one of the following: speech-language-hearing therapy, psychotherapy, and occupational therapy.

The data regarding intellectual assessment are described in Table 2. Although this variable was not used as an exclusion criterion, no child was classified as intellectually disabled (level V – percentile equal to or less than 5).

**Table 2:** Classification of intellectual estimation assessed using Raven's Progressive Matrices Test^(14)^

	**Superior**	**Above the mean**	**Mean**	**Below the mean**	**Discrepant** ^*^
**ASD group**	4 (15%)	9 (35%)	8 (31%)	4 (15%)	1 (4%)
**Control group**	2 (8%)	10 (38%)	8 (31%)	3 (12%)	3 (12%)

Data presented in the table by absolute frequency and percentage in parentheses

**Caption:** ASD = autism spectrum disorder. Source: The authors

* Due to a discrepancy > 2 in the stages of the Raven CPM test, it was not possible to calculate the subject’s intellectual capacity

The results regarding oral narrative discourse, assessed using the DNOI, are presented in Tables 3 and 4. Table 3 presents the data based on z-scores, while Table 4 presents the results according to the performance classification.

**Table 3:** Analysis of oral narrative discourse evaluated through the DNOI^(4)^, using Z-scores

	**Mean (SD)**		**Median (min; max)**		***P-Value***
	**ASD group**	**Control group**	**ASD group**	**Control group**	
**Partial retelling (essential information)**	-1.82 (1.6)	-0.53 (1.0)	-1.65 (-6.8; 0.6)	-0.69 (-2.1; 1.5)	0.002*
**Partial retelling (present information)**	-1.74 (1.4)	-0.79 (0.9)	-1.77 (-5.7; 0.4)	-0.95 (-2.2; 1.0)	0.01*
**Partial retelling (details)**	-1.08 (0.8)	-0.92 (0.6)	1.08 (-3.5; 0.2)	-1.08 (-2.2; 0.2)	0.69
**Total retelling**	-1.09 (1.8)	-0.45 (1.3)	-0.54 (-8.1; 1.4)	-0.38 (-3.9; 1.6)	0.22
**Oral comprehension**	-1.24 (1.9)	-0.4 (1.5)	-1.2 (-7.1; 1.8)	-0.4 (-3.6; 1.9)	0.14
**Inference**	-0.33 (1.0)	-0.32 (0.6)	-0.45 (-1.4; 3.2)	-0.46 (-1.24; 0.9)	0.86

Statistical analysis performed using the Mann-Whitney test (α = 0.05). The table presents data in Z-scores

**Caption:** ASD = autism spectrum disorder; SD = standard deviation; Min, Max = minimum and maximum values. Source: The authors

* Indicates statistical difference

**Table 4:** Analysis of oral narrative discourse assessed using the DNOI^(4)^ – percentage of children with adequate performance (average or above average classification)

	**ASD group**	**Control group**	***P-value***
**Partial retelling (essential information)**	7 (26.9%)	16 (61.5%)	**0.01** ^*^
**Partial retelling (present information)**	7 (26.9%)	15 (57.6%)	0.02*
**Partial retelling (details)**	10 (38.4%)	12 (46.1%)	0.57
**Total retelling**	16 (61.5%)	18 (69.2%)	55
**Total retelling (observations)**	16 (61.5%)	7 (26.9%)	0.37
**Oral comprehension**	13 (50%)	18 (69.2%)	0.15
**Inference**	21 (80.7%)	23 (88.4%)	0.44

Data presented in the table by absolute frequency and percentage in parentheses. Statistical analysis performed using the two-proportion z-test (α = 0.05)

**Caption:** ASD = autism spectrum disorder. Source: The authors

* Indicates a statistically significant difference

Regardless of the analysis method, the groups were statistically significantly different in the tasks of "essential information" and "present information" (both from the partial retelling task), with worse performance in the ASD group.

From a qualitative standpoint, most children in the ASD group did not perform satisfactorily in the partial retelling, due to the absence of essential information and the low amount of total information. On the other hand, more than half of the children in the ASD group performed satisfactorily in total retelling and story comprehension.

The qualitative analysis of complete retelling (represented only in Table 4, since it cannot be transformed into a z-score) addressed discursive behaviors during the narrative. The following were identified in the ASD group among the observation categories of the test: related intrusion (1; 4%), unrelated intrusion (4; 15%), tangential discourse (1; 4%), disregard for the chronology of events (2; 8%), imprecise references (1; 4%), decontextualized laughter (1; 4%), and omission of relation markers (2; 8%). In the CG, the following were observed: related intrusion (1; 4%), unrelated intrusion (1; 4%), tangential discourse (1; 4%), disregard for the chronology of events (6; 23%), and imprecise references (1; 4%).

Table 5 provides data on the morphosyntactic aspects of the discourse (number of nouns, verbs, adverbs, adjectives, prepositions, conjunctions, pronouns, and articles). The groups were statistically significantly different in nouns and prepositions, with superior performance in the CG.

**Table 5:** Analysis of morphosyntactic aspects, following the mean sentence value^(15)^, of the ASD and control groups

	**Mean (SD)**		**Median (min; max)**		***P- value***
	**ASD group**	**Control group**	**ASD group**	**Control group**	
**Nouns**	11.62 (5.9)	15 (6.6)	12 (0; 30)	16 (1; 26)	0.04*
**Verbs**	12.38 (5.6)	14.08 (4.9)	14 (0; 22)	15 (1; 26)	0.29
**Adverbs**	11.54 (10.2)	13.69 (8.6)	12 (0; 36)	12 (4; 32)	0.26
**Adjectives**	4.31 (3.3)	5.38 (4.3)	4 (0; 8)	4 (0; 16)	0.56
**Prepositions**	8.62 (7.16)	12.92 (7.4)	8 (0; 32)	16 (0; 28)	0.02*
**Conjunctions**	6.62 (5.76)	7.08 (6.3)	6 (0; 16)	6 (4; 16)	0.83
**Pronouns**	12.15 (11.18)	15.08 (8.8)	8 (0; 40)	16 (0; 36)	0.16
**Articles**	18.00 (10.1)	23.08 (10.7)	16 (0; 40)	24 (0; 40)	0.09
**Total**	127.08 (52.1)	154.69 (50.7)	126.5 (22; 225)	160 (40; 245)	0.08

Statistical analysis performed using the Mann-Whitney test (α = 0.05)

Caption: ASD = autism spectrum disorder; SD = standard deviation; Min, Max = minimum and maximum values. Source: The authors

* Indicates statistical difference

## DISCUSSION

This study aimed to analyze the characteristics of oral narrative in children with ASD who speak. It used the task of partial and complete story retelling, in which the evaluator read a story to the child, who then had to retell it orally. This task was carried out without the support of visual cues.

Narrative production requires a complex articulation between linguistic and cognitive skills. The narrator needs to access the desired vocabulary (access to the lexicon), organize sentences with appropriate syntax and grammar (syntax), and employ the relevant lexical elements (vocabulary). These processes depend on the integration between short-term phonological memory and oral discursive skill. In short, narrating implies mastery of linguistic aspects (e.g., pragmatics, semantics, syntax, morphology, and phonology) and cognitive aspects (e.g., memory, comprehension, monitoring, and inference)^(32)^. Given this complexity, individuals with atypical language development, such as those with ASD, are expected to have difficulties in narrative production^(17-18)^.

The study sample consisted mostly of boys (76.9%). Being male is considered a risk factor for ASD, as this diagnosis is more prevalent in men. The systematic review by Zeidan et al.^(26)^ demonstrated this by analyzing global prevalence estimates, considering geographic, ethnic, and socioeconomic factors. This difference can be partially explained by the fact that girls, especially those with milder clinical presentations, tend to manifest symptoms less evidently and adopt social camouflage strategies, which make identification and diagnosis of the disorder more difficult^(27)^.

Regarding socioeconomic level and maternal education, most children belonged to families of high socioeconomic status, with mothers having completed higher education. These conditions act as protective factors for child development^(28-30)^ and favor the early diagnosis of neurodevelopmental conditions and the search for therapies at the slightest sign of difficulty, due to greater knowledge about child development and better access to services^(28)^. On the other hand, children in unfavorable economic conditions face a greater risk of difficulties in various areas of development due to a lack of resources and stimuli^(31)^.

This study assessed the children's oral narrative skills through tasks involving partial retelling of a narrative (analyzing the presence of "essential information," "present information," and "details"), complete retelling of the same narrative, and discourse comprehension (by asking questions and requesting a title). The groups’ performances differed in the partial retelling task, specifically in the criteria of "essential information" and "present information," in both z-score comparison and performance classification; the ASD group performed worse. On the other hand, children with ASD and typically developing ones were not significantly different in the tasks of complete retelling, oral comprehension, and inference.

Study data indicate that the essential narrative elements were not adequately retold, which directly impacted the classification of the “total information”. A possible explanation for this result involves aspects such as behavioral rigidity and deficits in cognitive flexibility (characteristics often found in individuals with ASD) and difficulties related to attention and working memory. Both cognitive flexibility and working memory are part of a set of cognitive skills known as executive functions, which are responsible for processes such as planning, inhibitory control, monitoring, and adaptation to new situations^(33)^.

Regarding the first hypothesis, related to cognitive rigidity and difficulties in cognitive flexibility, it was observed that children with ASD frequently showed concern about the continuity of the story during the application of the task, trying to anticipate the events of the next paragraph instead of concentrating on the passage read, which hindered the retelling. This tendency, typical in children with autism, can negatively interfere with several tasks, as highlighted by Varanda and Fernandes^(34)^.

The second hypothesis refers to the difficulty in maintaining attention and memorizing sequences (working memory) – i.e., skills also related to executive functions, as well as cognitive flexibility. The study by Nayar et al.^(21)^ found that children with ASD presented reduced visual exploration of scenes from an illustrated book used as an eliciting stimulus. This behavior resulted in less visual attention to certain elements of the story, making them less salient or inefficiently encoded. The difficulty in perceiving and reporting the essential parts of the narrative seems to be associated with attentional deficits. This hypothesis is supported by the fact that the groups in the present study differed in the recall of essential items in a task with auditory input (read story), while in the study by Nayar et al.^(21)^, the task involved visual input, requiring the child to observe images and narrate the story.

Another point to discuss is the observations made by the evaluator during the complete retelling, despite the lack of statistical differences between the groups. The ASD group had related and unrelated intrusions, tangential discourse, disregard for the chronology of events, imprecise references, laughter, and omission of relation markers in the complete retelling task. Despite these observations, more than 60% of the children in both groups in this study were able to adequately retell the story.

These behaviors compromise narrative cohesion and coherence, central elements for the construction of interpretable and well-structured discourses^(13–15)^. The literature highlights that narrative production involves the integration of linguistic components (linked to the textual microstructure) and cognitive components (linked to the macrostructure), the latter being strongly dependent on executive functions such as inhibitory control, planning, organization, and working memory^(11-12,26)^. These functions allow the child to maintain thematic focus, organize the logical and temporal sequence of events, and understand their causal relationships — skills that are frequently impaired in individuals with ASD^(3,17-21)^.

The narrative behavior of children with ASD, such as deviations from the main storyline or disconnected speech, may reflect difficulties in the overall discourse planning and in monitoring their own production, processes mediated by executive functions^(26-27)^. As discussed, textual coherence develops gradually, being refined with the maturation of linguistic, cognitive, and discursive skills^(14)^. Although more than 60% of the children in both groups could adequately retell the story, the qualitative analysis of the productions reveals important nuances that are not captured by quantitative measures alone, but that directly impact the clarity, organization, and interpretability of the narrative text.

More than 50% of the children in both groups performed adequately in narrative comprehension. These findings are consistent with previous studies that indicate that children and adolescents with ASD, without intellectual disability, can have levels of comprehension similar to those of their typically developing peers when listening to narratives^(22)^.

The analysis of morphosyntactic performance of the discourse (elements that relate to microstructural aspects) showed that the CG used more syntactic elements, producing narratives with more elaborate and extensive sentences. Morphologically, there was a difference in the use of nouns and prepositions, with a greater occurrence of both morphological classes in the discourse of the CG. Previous studies report that participants with ASD produced shorter sentences, with less complex syntactic structures, alterations in prosodic aspects, incorrect pronoun use, and uncommon words, unlike typically developing children^(5,18,22,35)^.

A 2022 study^(36)^ indicated that morphosyntactic alterations in children with ASD occur mainly in those who have a diagnosis of language disorder as an ASD specifier, regardless of associated intellectual disability. However, some morphosyntactic errors may result from general difficulties in social interaction and discourse comprehension, without an underlying language impairment. These subtle errors were observed in the narratives of the children evaluated in this study.

These results corroborate the literature that describes oral narrative as a complex and progressively developing linguistic skill, involving both macrostructural and microstructural aspects^(13-15)^. The less use of morphosyntactic elements by children with ASD may be related to difficulties observed at earlier linguistic levels, such as semantic and pragmatic aspects, which are frequently altered in this group^(6,10,16)^. Such alterations may compromise the construction of more elaborate sentences, affecting textual cohesion and, consequently, the clarity and fluency of the narrative. The lower occurrence of nouns and prepositions in the narratives of the ASD group, for example, may indicate limitations in thematic expansion and in the spatial or temporal organization of narrated events, fundamental components for the progression of discourse. Furthermore, as described by previous studies^(5,18,22,35)^, subjects with ASD tend to produce shorter, less complex sentences with atypical use of pronouns, which was also subtly verified in the productions analyzed in this study.

It cannot be overlooked that children with ASD have highly heterogeneous development; some have inconsistent patterns of growth, stagnation, and regression in assessments^(37)^. Most children in this study were in therapy with speech-language-hearing pathologists and other interdisciplinary areas, demonstrating the direct influence of specialized intervention on the stimulation and development of general skills. Nonetheless, the groups in this study had homogeneous cognitive functioning; no child was identified with cognitive deficits, which proved to be of great importance for the analysis of the results, as there was no interference from cognitive capacity as a possible cause of the narrative deficits.

The limitations of this study encompass the exclusion of children with ASD from unfavorable sociodemographic conditions, such as difficulty accessing therapies, low socioeconomic status, and low maternal education levels, which can negatively impact linguistic and cognitive performance. Further studies with this population should analyze the impact of these variables on the development of children with ASD and preserved intellectual abilities. A methodological limitation of this study is that it used a task with exclusively auditory input, through storytelling and retelling, unlike most previous studies, which used visual stimuli (such as pictures or image sequences) to elicit oral narrative in individuals with ASD. This type of task requires additional skills, such as working memory – phonological loop, sustained attention, and discursive organization without visual support, which may have influenced the participants' performance, especially in the ASD group. This particularity limits direct comparability with other findings in the literature but reinforces the originality of the approach.

## CONCLUSION

Schoolchildren with ASD, with average or above-average intellectual abilities and from a high socio-economic-cultural family environment, with opportunities for early multidisciplinary treatment, present oral narrative discourse similar to that of typical children in the same sociodemographic conditions. Most of these schoolchildren with ASD can satisfactorily retell a story and understand a story told to them.

Children with ASD show impairments/lower performance in specific variables, such as partial retelling (essential and present information), and the morphosyntactic structure of their discourse differs only in the use of nouns and prepositions (which they use less) when compared to typically developing children.

Therefore, even in adverse neurodevelopmental conditions, as in the case of ASD, early diagnosis/stimulation, multidisciplinary follow-up, and adequate environmental conditions (family, school, and economic) can favor the child's linguistic development and efficiency.
