# Supplementary material for: Oral narrative discourse in schoolchildren with Autism Spectrum Disorder
Source: Codas. 2026 Apr 10;38(2):e20250042. doi: 10.1590/2317-1782/e20250042en (PMC13075842; doi:10.1590/2317-1782/e20250042en)
Supplement: Translation

# Translation: Discurso narrativo oral em escolares com Transtorno do Espectro Autista

**DOI:** 10.1590/2317-1782/e20250042pt

**Authors:** Luísa Stefano Santos, Patrícia Aparecida Zuanetti, Julia Branco Zulian, Matheus Francoy Alpes, Angela Cristina Pontes-Fernandes, Marisa Tomoe Hebihara Fukuda

**Affiliations:** 1 Departamento de Psicologia, Faculdade de Filosofia, Ciências e Letras de Ribeirão Preto – FFCLRP, Universidade de São Paulo – USP - Ribeirão Preto (SP), Brasil.; 2 Hospital das Clínicas de Ribeirão Preto, Faculdade de Medicina de Ribeirão Preto, Universidade de São Paulo – USP - Ribeirão Preto (SP), Brasil.; 3 Departamento de Formação Específica de Fonoaudiologia, Instituto de Saúde de Nova Friburgo, Universidade Federal Fluminense – INSF/UFF - Nova Friburgo (RJ), Brasil.; 4 Departamento Ciências da Saúde, Faculdade de Medicina de Ribeirão Preto, Universidade de São Paulo – USP - Ribeirão Preto (SP), Brasil.

**Keywords:** Transtorno do Espectro Autista, Narrativa, Linguagem Infantil, Desenvolvimento Infantil, Fatores de Proteção

## Abstract

**Objetivo:**

Analisar as características do discurso narrativo oral de crianças diagnosticadas com Transtorno do Espectro Autista (TEA).

**Método:**

Participaram escolares com idades entre 6 e 10 anos, divididos em dois grupos: G-TEA (n=26), composto por crianças com diagnóstico de TEA e oralizadas; G-Controle (n=26), formado por crianças com histórico de desenvolvimento típico. Dados sociodemográficos foram coletados por meio de questionários aplicados aos responsáveis, e os escolares foram avaliados quanto à estimativa intelectual e ao discurso narrativo oral (reconto parcial, reconto total e compreensão da história; a história foi lida para o escolar).

**Resultados:**

Os escolares pertenciam às classes socioeconômicas A/B, e a maioria das mães tinham nível superior completo. Quanto à estimativa intelectual, não houve escolares classificados como intelectualmente deficientes. No instrumento de narrativa oral, observou-se diferença entre os grupos na prova de reconto parcial, com pior desempenho do G-TEA. Não houve diferença no reconto total e na compreensão oral. Em relação à morfossintaxe utilizada na construção das frases narrativas, identificou-se diferença no uso de substantivos e preposições, com pior desempenho do G-TEA. Não foram observadas diferenças significativas no uso de outras categorias morfossintáticas.

**Conclusão:**

Escolares com TEA, em condições sociodemográficas favoráveis, apresentam desempenho narrativo oral semelhante ao de crianças típicas, com diferenças pontuais.

## INTRODUÇÃO

A narrativa oral é uma forma de comunicação intencional e de pensamento discursivo planejado para compartilhar experiências ordenadas no tempo, com causalidade e coerência^(1)^. Tal habilidade tem papel crucial para a organização da experiência, facilitando a interação social e a construção do conhecimento. É considerada uma habilidade cognitiva e linguística complexa^(2-3)^, que demanda a integridade das funções executivas^(4-5)^ e o domínio de habilidades semânticas e pragmáticas^(6)^.

Ainda que a definição de narrativa seja controversa, há consenso geral de que as narrativas implicam uma referência a sequência de eventos no tempo^(7-8)^. O discurso narrativo pode ser definido, então, como tarefa cognitiva complexa, que envolve a integração de informações, habilidades cognitivas, e uso do conhecimento acumulado sobre o mundo^(9)^. Narrativa, em outras palavras, seria a capacidade de pensar, comunicar e compartilhar experiências^(10)^.

A produção da narrativa envolve a integração de dois componentes principais: um linguístico e outro cognitivo. O componente linguístico, vinculado à microestrutura da narrativa, abrange aspectos como fonologia, morfossintaxe e semântica, sendo responsável pelo conteúdo da história. Já o componente cognitivo, relacionado à macroestrutura, envolve funções cognitivas de ordem superior, por exemplo as funções executivas, como a capacidade de inibir, planejar e organizar, além da manutenção do tema, da sequência lógica e temporal dos eventos e da compreensão das relações causais entre eles^(11-12)^. Esses elementos interagem para garantir a coesão e a coerência do discurso narrativo^(13)^.

O desenvolvimento da coerência textual é um processo gradual que reflete a maturação das habilidades linguísticas, cognitivas e discursivas da criança. À medida que avança em seu repertório linguístico e em sua capacidade de organizar pensamentos e ideias, a criança passa a estruturar narrativas de forma mais coesa, com maior conexão entre os eventos e clareza na progressão temática. Pesquisadores da área têm descrito esse processo como uma trajetória que pode ser observada por meio de níveis de desempenho, que indicam desde narrativas desorganizadas, com dificuldades na manutenção do tema e dos personagens, até produções bem estruturadas, com eventos logicamente encadeados e desfechos compatíveis com a trama desenvolvida. Essa perspectiva permite compreender e avaliar a coerência narrativa como uma habilidade em desenvolvimento, que se refina progressivamente ao longo da infância^(14)^. Instrumentos voltados para a avaliação dos aspectos macroestruturais da narrativa analisam elementos como cenário, tema, desenvolvimento do enredo, conflitos e sua resolução^(13-14)^.

Paralelamente ao desenvolvimento da coerência, a coesão textual desempenha um papel essencial na organização da narrativa, referindo-se aos mecanismos linguísticos que conectam as partes do texto entre si. A coesão corresponde à microestrutura da narrativa, formada por recursos como conectivos, pronomes, repetições e substituições lexicais que contribuem para a continuidade e o encadeamento das ideias. O uso apropriado desses elementos permite que o texto se apresente de forma fluida, facilitando a compreensão global e reforçando sua coerência^(15)^. Embora a coesão, por si só, não garanta a coerência textual, há uma estreita relação entre ambas, uma vez que os recursos coesivos funcionam como ferramentas linguísticas fundamentais para a construção de narrativas interpretáveis e bem estruturadas^(14)^. Os aspectos microestruturais são analisados por meio de medidas como o número total de palavras, variedade de vocábulos, uso de conjunções, unidades comunicativas, diversidade lexical, entre outros recursos linguísticos ^(13)^.

Seja no processo de compreensão ou de produção, a narrativa oral é um dos níveis mais elaborados da linguagem, e por isso, é esperado que indivíduos com alterações em níveis anteriores — como no desenvolvimento de aspectos linguísticos, tais como o semântico, pragmático ou outros - apresentem prejuízos em sua execução de forma efetiva^(6,10)^.

Indivíduos com transtornos do espectro autista tem como alteração base déficits persistentes em duas áreas principais: dificuldades em habilidades de comunicação social (aspecto pragmático da linguagem e cognição social) em múltiplos contextos e; padrões restritos e repetitivos de comportamento, interesses ou atividades, hipossensibilidade ou hipersensibilidade a estímulos sensoriais, como sons ou texturas e; estereotipias^(16)^. Além dessas características de base, crianças com TEA também podem apresentar alterações linguísticas e cognitivas mais globais como possíveis especificadores^(16)^.

A narrativa oral, geralmente eliciada por estímulo visual, tem sido estudada em indivíduos com TEA^(3,5,17-23)^, porém, os achados apresentados são variados, e por vezes inconsistentes. A diversidade nos achados ocorre devido à diversidade das amostras avaliadas (níveis variados de gravidade do TEA; associação a outras comorbidades tais como a deficiência intelectual; idade da amostra, alteração dos critérios diagnósticos para o TEA nos últimos anos, dentre outras variáveis).

Em geral estudos identificaram prejuízos nas habilidades de compreensão e produção de narrativa oral^(17-18)^; dificuldade em identificar, compreender e descrever as emoções dos personagens, interações dialógicas menos frequentes entre os personagens da história, prejuízos na compreensão de metáforas e metonímias^(19-20)^; velocidade de fala reduzida e maior número de hesitações^(3)^; narrativas mais curtas e gramaticalmente menos complexas^(5)^; omissão de componentes-chave da história^(21)^; melhor desempenho na produção de narrativas quando utilizadas sequências de figuras, em vez de uma única figura^(22)^, dentre outros achados. Mas, ao mesmo tempo, os mesmos estudos^(3,5,22)^ observaram, em outras variáveis analisadas, desempenho semelhante entre crianças típicas e crianças com TEA, tais como número de palavras relacionadas às relações temporais, número de “estados mentais produzidos”, compreensão e produção da história e outras variáveis.

Dada a relevância das narrativas para o desenvolvimento social e comunicativo e os desafios específicos enfrentados por indivíduos com TEA, estudar essa habilidade nesse grupo é fundamental. Tal investigação não apenas proporciona uma compreensão mais detalhada dos impactos do TEA na comunicação, mas também oferece subsídios para o desenvolvimento de estratégias de intervenção que visem promover habilidades narrativas mais eficazes, contribuindo para o fortalecimento das interações sociais e a melhoria da qualidade de vida dos indivíduos com TEA e suas famílias. O objetivo deste estudo foi analisar as características do discurso narrativo de crianças diagnosticadas com Transtorno do Espectro Autista (TEA).

## MÉTODO

### Modelo de estudo e considerações éticas

Trata-se de um estudo observacional do tipo transversal e foi realizado de acordo com as normativas da Comissão Nacional de Ética em Pesquisa (CONEP), submetido à avaliação pelo Comitê de Ética em pesquisa da FFCLRP - USP, e aprovado (CAAE nº 40972920.5.0000.5407). Todos os responsáveis pelas crianças avaliadas foram informados sobre o caráter da pesquisa, sendo orientados a assinar o Termo de Consentimento Livre e Esclarecido (TCLE).

### Seleção da amostra e local de coleta

O número amostral foi estabelecido por conveniência, baseado nas estimativas de sujeitos disponíveis nos locais de coleta (crianças que se enquadram nos critérios de inclusão do presente). As crianças do G-TEA foram recrutadas em uma clínica multidisciplinar especializada no atendimento de crianças com TEA, na qual frequentavam algum tipo de intervenção terapêutica. Cerca de 50 pacientes da clínica, com diagnóstico de TEA, nas idades de 6 a 10 anos, que correspondiam aos critérios de inclusão, foram convidados a participar do estudo, e apenas 26 responsáveis autorizaram a participação de seus filhos.

As crianças de G-Controle foram selecionadas em uma escola da rede particular de ensino, de modo que suas idades e sexos fossem pareadas ao Grupo Estudo. Para a seleção do grupo controle, a escola forneceu a lista de alunos matriculados, para seleção das crianças que correspondiam aos critérios da pesquisa e, para que as idades e sexos fossem pareadas. Caso houvesse mais de uma criança possível, houve um sorteio para saber qual criança seria convidada primeiramente para participar da pesquisa, caso não houvesse o interesse do responsável, a segunda criança pareada era convidada.

Foram adotados os seguintes critérios de inclusão: crianças com idade entre 6 e 10 anos. Para o grupo com Transtorno do Espectro Autista (G-TEA), as crianças deveriam apresentar diagnóstico médico e multidisciplinar de TEA, conforme os critérios do DSM-5-TR^(16)^, além de serem oralizadas, isto é, produzir frases simples e complexas com inteligibilidade, estrutura morfossintática adequada e sem alterações fonológicas moderadas ou graves. No grupo controle (G-Controle), foram incluídas apenas crianças que não estivessem inseridas em programas de educação especial ou com adaptações curriculares/programa de ensino individualizado, o que exclui aquelas com diagnóstico de TEA, deficiência intelectual, síndromes ou outras condições do neurodesenvolvimento.

Os critérios de exclusão foram: recusa, não realização ou interrupção na aplicação dos instrumentos. Além disso, para o G-Controle, foram excluídas crianças com qualquer diagnóstico médico ou multidisciplinar de transtorno do neurodesenvolvimento e/ou em uso de medicação com efeitos cognitivos. Ressalta-se que não foi considerado como critérios de inclusão/exclusão a presença de histórico de terapias multiprofissionais em ambos os grupos ou, estar em terapia não medicamentosa no momento da avaliação.

A amostra final deste estudo foi composta por 52 crianças com idades entre 6 e 10 anos, divididos em:

- G - TEA (n=26; idade média 7,2; DP: 1,3; 20 do sexo masculino), composto por crianças com Transtorno do Espectro Autista (TEA) e oralizados;
- G - Controle (n=26; idade média 7,1; DP: 1,3; 20 do sexo masculino), composto por crianças com desenvolvimento típico, pareados por idade e sexo ao G1.

### Instrumentos e procedimentos de coleta

Tanto na clínica, como na escola, as crianças foram avaliadas individualmente, em salas com iluminação adequada e sem ruído externo competitivo que pudessem comprometer o seu desempenho. Estavam presentes na sala somente a criança e a examinadora. Com exceção da avaliação da estimativa intelectual, a qual foi realizada por uma psicóloga, todos os outros instrumentos foram aplicados por profissional fonoaudiólogo, e gravados, através do software Audacity®, com ajuda de um microfone para que a resposta da criança pudesse ser posteriormente analisada.

Com os pais/responsáveis, foram utilizados os seguintes instrumentos:

- Entrevista inicial: breve questionário estruturado respondido pelos responsáveis, onde foi possível coletar informações referentes ao histórico da criança. Este tinha como objetivo aplicar/confirmar os critérios de inclusão/exclusão e para a caracterização da amostra.
- Critério de Classificação Econômica Brasil – ABEP^(23)^: instrumento que tem como objetivo classificar o nível socioeconômico da família do sujeito de pesquisa. Este questionário é respondido pelo responsável e consiste na quantificação de variáveis existentes na residência da família, grau de instrução do “chefe de família” e acesso a serviços públicos. De acordo com as respostas apresentadas, houve a pontuação e classificação do nível socioeconômico da família. Esta variável foi utilizada para caracterização da amostra.

Com as crianças, foram utilizados os seguintes instrumentos:

- Matrizes Progressivas Coloridas de Raven^(24)^: É uma medida não verbal de inteligência. A Escala Colorida é especificamente projetada para crianças, indivíduos com dificuldades de comunicação, ou aqueles com desenvolvimento atípico, por minimizar a influência de fatores linguísticos e culturais sobre o desempenho. O teste consiste em uma série de problemas visuais apresentados em formato de matrizes, nos quais o indivíduo deve identificar o padrão ou a lógica que rege a sequência e selecionar a alternativa correta para completá-la. A aplicação foi realizada de forma individual, seguindo as orientações técnicas do manual do teste. Os resultados deste instrumento foram neste estudo foram utilizados com o objetivo de caracterizar a amostra e, os dados foram apresentados em variável categórica (classificação do desempenho segundo normas do teste).
- Discurso Narrativo Oral – DNOI^(4)^: é um instrumento de avaliação que tem como objetivo avaliar a linguagem em nível discursivo, sendo uma ferramenta útil para avaliar sujeitos com dificuldades de linguagem e de disfunção executiva. Este é composto por três subtarefas: reconto parcial de uma narrativa (a criança foi informada de que ouviria uma história curta, e que após cada parágrafo, deveria recontar o que acabou de ouvir); reconto integral da mesma narrativa (o texto inteiro foi lido à criança, que, ao final, teve que recontar com suas palavras, a história) e; compreensão discursiva - literal e inferencial (solicitada a elaboração de um título, para a narrativa ouvida, assim como respostas, para perguntas a certa da história). Ressalta-se que essa tarefa não conta com apoio visual. Em todas as três subtarefas, o avaliador lê os parágrafos ou textos para as crianças e faz as perguntas oralmente. Da mesma forma, as respostas devem ser dadas oralmente.

A aplicação foi realizada de forma individual, seguindo as orientações técnicas do manual do teste. As respostas foram gravadas e posteriormente analisadas. Os resultados dos desempenhos quantitativos foram analisados tendo como base o cálculo do z-score (transformação do escore bruto segundo a idade e tipo de escola da criança, transformação sugerida pelo próprio instrumento) e, com base no z-escore, o desempenho da criança nas diversas tarefas também foi classificado em adequado (z-escore maior que -0,99) e alterado (z-escore igual ou menor a -1,0).

- Conteúdo morfossintático do discurso: na amostra de narrativa foram analisados os aspectos morfossintáticos conforme parâmetros descritos por meio da análise “Média do Valor da Frase”^(25)^. Para essa análise, considerou-se as cinco primeiras frases faladas pela criança, na prova de reconto integral do DNOI, as quais foram avaliadas e pontuadas de acordo com os elementos sintáticos e lexicais utilizados, levando em consideração sua complexidade: os substantivos e verbos, por serem considerados os primeiros a surgir na aquisição da linguagem e darem significado à frase, tiveram peso 2, ou seja, o número de substantivos e verbos utilizados pela criança foi multiplicado por 2; já os advérbios, adjetivos, preposições, conjunções, pronomes e artigos, tiveram peso 4, ou seja, para cada uma das palavras emitidas referentes a essas categorias, foi multiplicado por 4. Os resultados dos desempenhos numéricos apresentados pelas crianças foram analisados tendo como base o peso correspondente a cada um dos elementos sintáticos (2 ou 4, conforme descrito acima).

### Análise estatística dos dados

Utilizou-se estatística descritiva para a caracterização dos grupos e, para a inferência, o Teste Igualdade de Proporções entre Duas Amostras (comparação de alguns dados da anamnese e das variáveis qualitativas/classificações de desempenho nos diversos testes utilizados) e, o teste Mann – Whitney (comparação das variáveis quantitativas, ou seja, do Z-Escore). Para ambos os testes, o nível de significância adotado foi de α = 0,05.

## RESULTADOS

Em relação à escolaridade do G-TEA, 20 crianças (76,9%) estavam matriculadas no ensino fundamental I (sendo 13 no 1º ano, 3 no 2º ano, 3 no 3º ano e 2 no 5º ano). No G-Controle, 25 crianças (96,1%) frequentavam o ensino fundamental (14 no 1º ano, 5 no 2º ano, 4 no 3º ano e 2 no 4º ano). Em ambos os grupos, os responsáveis relataram dois casos de repetência.

Na Tabela 1, são apresentadas as variáveis sociodemográficas, incluindo escolaridade materna e classificação socioeconômica. Com relação à escolaridade das mães, houve predomínio do ensino superior completo em ambos os grupos. Quanto à classificação socioeconômica, observou-se maior concentração nas classes mais altas (A e B).

**Tabela 1:** Caracterização da amostra em relação à escolaridade materna e classificação socioeconômica (Critério de Classificação Econômica Brasil - ABEP)^(17)^

		**G - TEA**	**G – Controle**
		**Freq Ab. (%)**	**Freq Ab. (%)**
**Escolaridade Materna**	Ensino Fundamental II	1 (3,8%)	0 (0%)
	Ensino Médio	2 (7,6%)	2 (7,6%)
	Ensino Superior Incompleto	4 (15,3%)	1 (3,8%)
	Ensino Superior Completo	16 (61,5%)	15 (57,6%)
	Pós-graduação	3 (11,5%)	8 (30,7%)
**Classificação Econômica**	Classe A	9 (34,6%)	13 (50%)
	Classe B1	6 (23%)	5 (19,2%)
	Classe B2	10 (38,4%)	6 (23%)
	Classe C1	1 (3,8%)	2 (7,6%)

**Legenda:** TEA = Transtorno do Espectro Autista; Freq Ab. (%) = Dados apresentados na tabela por Frequência Absoluta e Porcentagem entre parênteses. Fonte: Autores

A média de idade ao diagnóstico no G-TEA foi de 4,0 anos (DP: 1,7), com idade mínima de dois anos e máxima de oito anos. Esses dados revelam uma ampla variação na idade de diagnóstico, indicando que algumas crianças foram identificadas e encaminhadas para intervenção ainda na primeira infância, enquanto outras, possivelmente com manifestações mais leves do TEA, receberam diagnóstico mais tardiamente.

Quanto ao histórico de intervenção terapêutica, observou-se maior prevalência no G-TEA. Todas as crianças deste grupo (n = 26; 100%) apresentavam histórico de terapia (fonoaudiologia, psicologia e/ou terapia ocupacional). No G-Controle, apenas 6 crianças (23%) relataram algum tipo de intervenção. Destaca-se que, no momento da coleta, todas as crianças do G-TEA estavam em acompanhamento terapêutico ativo (em pelo menos uma das áreas: fonoterapia, psicoterapia e/ou terapia ocupacional).

Os dados referentes à estimativa intelectual encontram-se descritos na Tabela 2. Embora essa variável não tenha sido utilizada como critério de exclusão, nenhuma criança foi classificada como intelectualmente deficiente (nível V – percentil igual ou inferior a 5).

**Tabela 2:** Classificação da estimativa intelectual avaliado por meio do Teste das Matrizes Progressivas de Raven^(14)^

	**Superior**	**Acima da média**	**Média**	**Abaixo da média**	**Discrepante** ^*^
**G – TEA**	4 (15%)	9 (35%)	8 (31%)	4 (15%)	1 (4%)
**G - Controle**	2 (8%)	10 (38%)	8 (31%)	3 (12%)	3 (12%)

Dados apresentados na tabela por Frequência Absoluta e Porcentagem entre parênteses

**Legenda:** TEA = Transtorno do Espectro Autista. Fonte: Autores

* Devido à discrepância > 2 nas etapas do teste CPM – RAVEN, não foi possível calcular a capacidade intelectual do sujeito avaliado

Os resultados referentes ao discurso narrativo oral, avaliados por meio do instrumento DNOI, são apresentados nas Tabelas 3 e 4. A Tabela 3 traz os dados com base nos escores-z, enquanto a Tabela 4 apresenta os resultados conforme a classificação de desempenho.

**Tabela 3:** Análise do discurso narrativo oral avaliado através do DNOI^(4)^; utilizando os valores do *Z-escore*

	**Média (DP)**		**Mediana (min; máx)**		***P-Valor***
	**G – TEA**	**G - Controle**	**G - TEA**	**G - Controle**	
**Reconto parcial (inf. essenciais)**	-1,82 (1,6)	-0,53 (1,0)	-1,65 (-6,8; 0,6)	-0,69 (-2,1; 1,5)	**0,002** ^*^
**Reconto parcial (inf. presentes)**	-1,74 (1,4)	-0,79 (0,9)	-1,77 (-5,7; 0,4)	-0,95 (-2,2; 1,0)	0,01*
**Reconto parcial (detalhes)**	-1,08 (0,8)	-0,92 (0,6)	1,08 (-3,5; 0,2)	-1,08 (-2,2; 0,2)	0,69
**Reconto total**	-1,09 (1,8)	-0,45 (1,3)	-0,54 (-8,1; 1,4)	-0,38 (-3,9; 1,6)	0,22
**Compreensão oral**	-1,24 (1,9)	-0,4 (1,5)	-1,2 (-7,1; 1,8)	-0,4 (-3,6; 1,9)	0,14
**Inferência**	-0,33 (1,0)	-0,32 (0,6)	-0,45 (-1,4; 3,2)	-0,46 (-1,24; 0,9)	0,86

Análise estatística realizada através do teste de Mann - Whitney (α = 0,05). Dados apresentados na tabela são os valores do Z-Escore

**Legenda:** TEA = Transtorno do Espectro Autista; DP = Desvio Padrão; Min, Máx = valor mínimo e valor máximo. Fonte: Autores

* Indica diferença estatística

**Tabela 4:** Análise do discurso narrativo oral avaliado através do DNOI^(4)^ – porcentagem de crianças com desempenho adequado (classificação média ou acima da média)

	**G - TEA**	**G - Controle**	***P - valor***
**Reconto parcial (Inf. essenciais)**	7 (26,9%)	16 (61,5%)	0,01*
**Reconto parcial (Inf. presentes)**	7 (26,9%)	15 (57,6%)	0,02*
**Reconto parcial (detalhes)**	10 (38,4%)	12 (46,1%)	0,57
**Reconto total**	16 (61,5%)	18 (69,2%)	55
**Reconto total (observações)**	16 (61,5%)	7 (26,9%)	0,37
**Compreensão oral**	13 (50%)	18 (69,2%)	0,15
**Inferência**	21 (80,7%)	23 (88,4%)	0,44

Dados apresentados na tabela por Frequência Absoluta e Porcentagem entre parênteses. Análise estatística realizada através do Teste de Igualdade de Proporções (α = 0,05)

**Legenda:** TEA = Transtorno do Espectro Autista. Fonte: Autores

* Indica diferença estatisticamente significante

Independentemente da forma de análise, nas tarefas de “informações essenciais” e “informações presentes” — ambas da etapa de reconto parcial —, houve diferença estatisticamente significativa entre os grupos, com pior desempenho no G-TEA.

Do ponto de vista qualitativo, observou-se que a maioria das crianças do G-TEA não apresentou desempenho satisfatório no reconto parcial, caracterizado pela ausência de informações essenciais e pela baixa quantidade de informações totais. Por outro lado, nas etapas de reconto total e compreensão da história, mais da metade das crianças do G-TEA apresentou desempenho satisfatório.

Na análise qualitativa do reconto integral — variável representada apenas na Tabela 4, uma vez que não é passível de transformação em escore-z — foram analisados os comportamentos discursivos durante a narrativa. Dentre as categorias de observação do teste, no G-TEA, identificaram-se: intrusão relacionada (1; 4%), intrusão não relacionada (4; 15%), discurso tangencial (1; 4%), desrespeito à cronologia dos eventos (2; 8%), referências imprecisas (1; 4%), risos descontextualizados (1; 4%) e omissão de marcadores de relação (2; 8%). No G-Controle, observaram-se: intrusão relacionada (1; 4%), intrusão não relacionada (1; 4%), discurso tangencial (1; 4%), desrespeito à cronologia dos eventos (6; 23%) e referências imprecisas (1; 4%).

As informações sobre os aspectos morfossintáticos do discurso — número de substantivos, verbos, advérbios, adjetivos, preposições, conjunções, pronomes e artigos — encontram-se na Tabela 5. Verificou-se diferença estatisticamente significativa entre os grupos nas categorias de substantivos e preposições, com desempenho superior do G-Controle.

**Tabela 5:** Análise dos aspectos morfossintáticos, seguindo a Média do Valor da Frase^(15)^, de G1 e G2

	**Média (DP)**		**Mediana (min; máx)**		***P- valor***
	**G - TEA**	**G - Controle**	**G - TEA**	**G - Controle**	
**Substantivo**	11,62 (5,9)	15 (6,6)	12 (0; 30)	16 (1; 26)	0,04*
**Verbo**	12,38 (5,6)	14,08 (4,9)	14 (0; 22)	15 (1; 26)	0,29
**Advérbio**	11,54 (10,2)	13,69 (8,6)	12 (0; 36)	12 (4; 32)	0,26
**Adjetivo**	4,31 (3,3)	5,38 (4,3)	4 (0; 8)	4 (0; 16)	0,56
**Preposição**	8,62 (7,16)	12,92 (7,4)	8 (0; 32)	16 (0; 28)	0,02*
**Conjunções**	6,62 (5,76)	7,08 (6,3)	6 (0; 16)	6 (4; 16)	0,83
**Pronomes**	12,15 (11,18)	15,08 (8,8)	8 (0; 40)	16 (0; 36)	0,16
**Artigos**	18,00 (10,1)	23,08 (10,7)	16 (0; 40)	24 (0; 40)	0,09
**Total**	127,08 (52,1)	154,69 (50,7)	126,5 (22; 225)	160 (40; 245)	0,08

Análise estatística realizada através do teste de Mann - Whitney (α = 0,05)

**Legenda:** TEA = Transtorno do Espectro Autista; DP = Desvio Padrão; Min, Máx = valor mínimo e valor máximo. Fonte: Autores

* Indica diferença estatística

## DISCUSSÃO

O presente estudo teve como objetivo analisar as características da narrativa oral em crianças com Transtorno do Espectro Autista (TEA) oralizadas. Para tanto, foi utilizada a tarefa de reconto parcial e integral de histórias, na qual o avaliador lia uma história para a criança, que, em seguida, deveria recontá-la oralmente. Ressalta-se que essa tarefa foi realizada sem o apoio de pistas visuais.

A produção de narrativas exige uma articulação complexa entre habilidades linguísticas e cognitivas. O narrador precisa acessar os vocábulos desejados (acesso ao léxico), organizar as sentenças de forma sintática e gramaticalmente adequada (sintaxe), e empregar os elementos lexicais pertinentes (vocabulário). Esses processos dependem da integração entre a memória fonológica de curto prazo e a habilidade discursiva oral. Em síntese, narrar implica o domínio de aspectos linguísticos — como pragmática, semântica, sintaxe, morfologia e fonologia — e cognitivos, como memória, compreensão, monitoramento e inferência^(32)^. Diante dessa complexidade, é esperado que indivíduos com desenvolvimento atípico da linguagem, como aqueles com TEA, apresentem dificuldades na produção narrativa^(17-18)^.

A amostra deste estudo foi composta majoritariamente por meninos (76,9%). O sexo masculino é considerado um fator de risco no TEA, sendo que esse diagnóstico é mais prevalente em homens, como evidenciado pela revisão sistemática de Zeidan et al.^(26)^, que analisou estimativas globais de prevalência considerando fatores geográficos, étnicos e socioeconômicos. Essa diferença pode ser parcialmente explicada pelo fato de que meninas, especialmente aquelas com apresentações clínicas mais leves, tendem a manifestar sintomas de forma menos evidente e a adotar estratégias de camuflagem social, o que dificulta a identificação e o diagnóstico do transtorno^(27)^.

Quanto ao nível socioeconômico e escolaridade materna, a maioria das crianças pertencia a famílias de alto nível socioeconômico, com mães com ensino superior completo. Essas condições atuam como fatores de proteção ao desenvolvimento infantil^(28-30)^ e favorecem o diagnóstico precoce de condições do neurodesenvolvimento e a busca por terapias ao menor sinal de dificuldade, devido ao maior conhecimento sobre desenvolvimento infantil e melhor acesso a serviços^(28)^. Por outro lado, crianças em condições econômicas desfavoráveis enfrentam maior risco de dificuldades em várias áreas do desenvolvimento devido à falta de recursos e estímulos^(31)^.

As habilidades narrativas orais das crianças, neste presente estudo, foram avaliadas por meio de tarefas de: reconto parcial de uma narrativa (no qual era analisado a presença de “informações essenciais”, “informações presentes” e “detalhes”), reconto integral (da mesma narrativa) e compreensão discursiva (no qual foram realizadas perguntas e solicitado a elaboração de um título). Observou-se que, na tarefa de reconto parcial, especificamente nos critérios de “informações essenciais” e “informações presentes”, houve diferença de desempenho entre os grupos — tanto na comparação dos escores-z quanto na classificação do desempenho —, com desempenho inferior no grupo com TEA (G-TEA). Por outro lado, nas tarefas de reconto total, compreensão oral e inferência, não foram encontradas diferenças significativas entre as crianças com TEA e aquelas com desenvolvimento típico.

Os dados apresentados indicam que os elementos narrativos essenciais não foram recontados de forma adequada, o que impactou diretamente a classificação das “informações totais”. Uma possível explicação para esse resultado envolve aspectos como rigidez comportamental e déficits em flexibilidade cognitiva — características frequentemente observadas em indivíduos com TEA —, além de dificuldades relacionadas à atenção e à memória de trabalho. Tanto a flexibilidade cognitiva quanto a memória de trabalho fazem parte de um conjunto de habilidades cognitivas conhecido como funções executivas, que são responsáveis por processos como planejamento, controle inibitório, monitoramento e adaptação a novas situações^(33)^.

No que se refere à primeira hipótese, relacionada à rigidez cognitiva e dificuldades em flexibilidade cognitiva, foi observado que, durante a aplicação da tarefa, as crianças com TEA frequentemente se mostravam preocupadas com a continuidade da história, tentando antecipar os eventos do próximo parágrafo em vez de se concentrarem no trecho lido, o que prejudicava o reconto. Essa tendência, típica em crianças com autismo, pode interferir negativamente em diversas tarefas, como destacado por Varanda e Fernandes^(34)^.

A segunda hipótese refere-se à dificuldade em manter a atenção e memorizar sequências (memória trabalho), ou seja, habilidades também relacionadas às funções executivas, assim como a flexibilidade cognitiva. No estudo de Nayar et al.^(21)^, observou-se que crianças com TEA apresentaram uma exploração visual reduzida das cenas de um livro ilustrado utilizado como estímulo eliciador. Esse comportamento resultou em menor atenção visual a determinados elementos da história, tornando-os menos salientes ou ineficientemente codificados. A dificuldade em perceber e relatar as partes essenciais da narrativa parece estar associada a déficits atencionais. Tal hipótese é sustentada pelo fato de que, em nosso estudo, a diferença entre os grupos foi observada na recordação de itens essenciais em uma tarefa com entrada auditiva (história lida), enquanto no estudo de Nayar et al.^(21)^, a tarefa envolvia entrada visual, exigindo que a criança observasse imagens e narrasse a história.

Outro dado a ser discutido, apesar de não ter tido diferença estatística entre os grupos, foram as “observações” realizadas pelo avaliador durante o reconto integral. Na tarefa de reconto integral foi observada no G-TEA, a ocorrência de intrusões (relacionadas e/ou não relacionadas), discurso tangencial, não respeito à cronologia dos eventos, referências imprecisas, risadas, e omissão de marcadores de relação. Apesar dessas “observações”, mais de 60% das crianças de ambos os grupos deste estudo conseguiram recontar adequadamente a história.

Esses comportamentos comprometem a coesão e a coerência da narrativa, elementos centrais para a construção de discursos interpretáveis e bem estruturados^(13–15)^. A literatura já destaca que a produção narrativa envolve a integração de componentes linguísticos (ligados à microestrutura textual) e cognitivos (ligados à macroestrutura), sendo estes últimos fortemente dependentes de funções executivas como controle inibitório, planejamento, organização e memória de trabalho^(11–12, 26)^. Essas funções permitem à criança manter o foco temático, organizar a sequência lógica e temporal dos eventos e compreender suas relações causais — habilidades frequentemente prejudicadas em indivíduos com TEA^(3,17–21)^.

O comportamento narrativo observado nas crianças com TEA, como desvios da linha principal da história ou falas desconectadas, pode refletir dificuldades no planejamento global do discurso e no monitoramento de sua própria produção, processos mediados por funções executivas^(26–27)^. Como discutido, o desenvolvimento da coerência textual é gradual e se refina com a maturação das habilidades linguísticas, cognitivas e discursivas^(14)^. Ainda que mais de 60% das crianças dos dois grupos tenham conseguido recontar adequadamente a história, a análise qualitativa das produções evidencia nuances importantes que não são captadas apenas por medidas quantitativas, mas que impactam diretamente a clareza, a organização e a interpretabilidade do texto narrativo.

No que se refere à compreensão da narrativa, observou-se que mais de 50% das crianças de ambos os grupos apresentaram desempenho adequado na atividade. Esses achados estão em consonância com estudos anteriores que indicam que crianças e adolescentes com TEA, sem deficiência intelectual, podem demonstrar níveis de compreensão semelhantes aos de seus pares com desenvolvimento típico ao ouvirem narrativas^(22)^.

Quando analisado o desempenho morfossintático do discurso, elementos estes que dizem respeito aos aspectos microestruturais, observou- se maior uso de elementos sintáticos pelo grupo controle que produziram frases mais elaboradas e extensas em sua narrativa. Morfologicamente, houve diferença no uso de substantivos e preposição, com maior ocorrência de ambas as classes morfológicas no discurso do grupo controle. Estudos anteriores descrevem que os enunciados produzidos pelos sujeitos com a condição de TEA eram mais curtos, apresentavam estruturas sintáticas menos complexas, alteração nos aspectos prosódicos, uso incorreto de pronomes e presença de palavras incomuns, diferentemente das crianças com desenvolvimento típico^(5,18,22,35)^.

Um estudo de 2022^(36)^ indicou que as alterações morfossintáticas em crianças com TEA ocorrem principalmente naquelas que apresentam o diagnóstico de transtorno de linguagem como especificador do TEA, independentemente da presença de deficiência intelectual associada. No entanto, alguns erros morfossintáticos podem resultar de dificuldades gerais na interação social e na compreensão do discurso, sem um comprometimento subjacente da linguagem. Esses erros sutis foram observados na narrativa das crianças avaliadas neste estudo.

Esses resultados corroboram a literatura que descreve a narrativa oral como uma habilidade linguística complexa e de desenvolvimento progressivo, que envolve tanto aspectos macroestruturais quanto microestruturais^(13-15)^. O uso reduzido de elementos morfossintáticos pelas crianças com TEA pode estar relacionado às dificuldades observadas em níveis linguísticos anteriores, como aspectos semânticos e pragmáticos, frequentemente alterados nesse grupo^(6,10,16)^. Tais alterações podem comprometer a construção de enunciados mais elaborados, afetando a coesão textual e, consequentemente, a clareza e fluidez da narrativa. A menor ocorrência de substantivos e preposições observada nas narrativas do grupo TEA, por exemplo, pode indicar limitações na expansão temática e na organização espacial ou temporal dos eventos narrados, componentes fundamentais para a progressão do discurso. Ainda, como descrito por estudos anteriores^(5,18,22,35)^, sujeitos com TEA tendem a produzir enunciados mais curtos, menos complexos e com uso atípico de pronomes, o que também foi verificado de forma sutil nas produções analisadas neste estudo.

Não se pode deixar de dizer que, as crianças com TEA também mostram alta heterogeneidade nas trajetórias de desenvolvimento, com algumas apresentando padrões inconsistentes de crescimento, estagnação e regressão nas avaliações^(37)^. A maioria das crianças deste estudo se encontravam em terapia fonoaudiológica e de outras áreas interdisciplinares, demonstrando a influência direta da intervenção especializada frente à estimulação e evolução de habilidades gerais. É importante ressaltar que no presente estudo os grupos foram homogêneos em relação ao funcionamento cognitivo, não tendo sido identificada crianças com déficit cognitivo, o que se mostrou de grande importância para a análise dos resultados obtidos, de modo que, não houvesse interferência da capacidade cognitiva como possível causa dos déficits narrativos.

As limitações deste estudo referem-se, primeiramente, à não inclusão de crianças com TEA em condições sociodemográficas desfavoráveis, como dificuldade de acesso a terapias, baixo nível socioeconômico e baixo nível de escolaridade materna — variáveis que podem impactar negativamente o desempenho linguístico e cognitivo. Sugere-se a realização de estudos com essa população, com o objetivo de analisar o impacto dessas variáveis no desenvolvimento de crianças com TEA, mas com estimativa intelectual preservada. Além disso, destaca-se como limitação metodológica o fato de que, diferentemente da maioria dos estudos prévios que utilizaram estímulos visuais (como figuras ou sequências de imagens) para elicitar a narrativa oral em indivíduos com TEA, o presente estudo utilizou uma tarefa com entrada exclusivamente auditiva, por meio de conto e reconto. Esse tipo de tarefa exige habilidades adicionais, como memória de trabalho – alça fonológica, atenção sustentada e organização discursiva sem suporte visual, o que pode ter influenciado o desempenho dos participantes, especialmente no grupo TEA. Tal particularidade limita a comparabilidade direta com outros achados da literatura, mas reforça a originalidade da abordagem adotada.

## CONCLUSÃO

Escolares com Transtorno do Espectro Autista, com estimativa intelectual na média ou acima/superior à média e provenientes de um ambiente familiar de alto nível sócio-econômico-cultural, com oportunidades de tratamento multiprofissional precoce, apresentam discurso narrativo oral semelhante a crianças típicas em mesmas condições sociodemográficas. A maioria destes escolares com TEA conseguem recontar satisfatoriamente uma história e compreender uma história que lhe é contada pelo outro.

As crianças com TEA apresentam prejuízos/desempenho inferior em variáveis pontuais, como no reconto parcial (informações essenciais e presentes) e, a estrutura morfossintática de seu discurso se difere apenas no uso de substantivos e preposições (utilizam menos estas categorias) quando comparado ao de crianças com desenvolvimento típico.

Assim sendo, mesmo em condições adversas do neurodesenvolvimento, como no caso do TEA, o diagnóstico/estimulação em idade precoce, o acompanhamento multiprofissional, e as condições ambientais adequadas (familiares, escolares e econômicas), podem favorecer o desenvolvimento e eficiência linguística da criança.
